# Comparison of the Electrocardiographic Features of Complete Left Bundle Branch Block in Patients with  Ischemic and Nonischemic Left Ventricular Dysfunction

**Published:** 2007-01-01

**Authors:** Bulent Deveci, Ozcan Ozeke, Mehmet Fatih Ozlu, Ozgul Malcok Gurel, Mehmet Timur Selcuk, Serkan Topaloglu, Orhan Maden, Kumral Ergun, Aytun Canga, Tumer Erdem Guler, Veli Kaya, Dursun Aras

**Affiliations:** 1 Yuksek Ihtisas Education and Research Hospital, Cardiology, Ankara, Turkey; 2 Meram Education and Research Hospital, Cardiology, Konya, Turkey; 3 Tokat Dr. Cevdet Aykan State Hospital, Cardiology, Tokat, Turkey

**Keywords:** left bundle branch block, ischemic left ventricular dysfunction

## Abstract

**Background:**

Differentiating ischemic (ILVD)  from nonischemic left ventricular dysfunction (NILVD) is important prognostically and therapeutically but might be difficult clinically. The differentiating role of electrocardiographic (ECG) features in the presence of left bundle-branch block (LBBB) is debatable on differentiating ILVD from NILVD.

**Objective:**

The present study assessed whether there is the role of certain ECG features in differentiating ILVD from NILVD in the presence of the complete LBBB.

**Methods and Results:**

Patients who had LBBB were divided into two groups based on the presence and type of  left ventricular dysfunction; (1) ILVD group (49 patients;  20 female; age: 65 ± 11 years) and (2) NILVD group (49 patients; 22 female; age: 59 ± 12 years), and numerous ECG features were compared. Most of these ECG features did not show any difference between the groups except for following ECG findings; the voltage of R wave in V6 were statistically higher in NILVD group compared ILVD group (p: 0.03); the depression of the ST-J point by more than 0.2 mV in V6 were also frequently observed in NILVD  group compared ILVD group (5/ 10%  vs 19/ 39% , p: 0.001); and  the notching in the ascending or descending limb of the S wave in V1-4 leads were more in ILVD group (18/ 36% vs 8/ 16% p: 0.03; 9/ 16%  vs  2/ 4%, p: 0.03, respectively).

**Conclusion:**

In the current study, although some ECG findings were found to be useful, ECG features in the presence of complete LBBB had poor value in differentiating ILVD from NILVD.

## Introduction

Left bundle branch block (LBBB) is often associated with underlying structural heart disease such as hypertension, idiopathic dilated or ischemic left ventricular dysfunction [1-4]. Differentiating ILVD from NILVD is important prognostically and therapeutically but might be difficult clinically [5]. In general, the noninvasive tests are not reliable in distinguishing left ventricular dysfunction related to coronary artery disease (CAD) or  nonischemic reasons [6]. In many centers, coronary angiography is routinely performed in all cases for this task despite increased costs and inherent risks associated with invasive cardiac catheterization [7]. The diagnostic and prognostic value of ECG information is being increasingly recognized. In the present study, we evaluated and compared the numerous electrocardiographic (ECG) features in patients with NILVD or ILVD in the presence of the complete LBBB.

## Methods

The files of all (2567) patients in the hospital's computer archives were reviewed to identify patients with LBBB. The medical records of those with LBBB were reviewed for clinical, ECG, echocardiographic (for LVEF) and angiocardiographic findings. Patients who had LBBB were divided into 2 groups based on the presence and type of left ventricular dysfunction: (1) ILVD group (if they had a history of old myocardial infarction, percutaneous coronary intervention, coronary artery bypass graft surgery or at least one major epicardial coronary artery with > or = 75% stenosis and left ventricular ejection fraction (LVEF)  < 40 % ); (2) NILVD group (if they had left ventricular dilation with global systolic dysfunction with LVEF < 40 % and  without a frank scar or aneurysm by echocardiography and absence of CAD). Cases of new-acute myocardial infarction; incomplete LBBB; non-supraventricular or electronic pacemaker rhythm were excluded. ECGs of patients were reviewed by one cardiologist blinded for the etiology of the left ventricular dysfunction. If the patient had many ECGs, the ECG at the day of the coronary angiography was used. ECG duration and voltage were manually measured.The ECG features evaluated are listed in Table 1.

We used the following definition of LBBB: 1) QRS of more than 0.125 milliseconds in the presence of normal sinus or supraventricular rhythm; 2) QS or rS complex in lead V1; 3) broad or notched R waves in leads V5 and V6 or an RS pattern 4), and R peak time ≥0.06 s absence of a Q wave in leads V5, V6, and I [7]. Left axis deviation was considered present when the mean frontal QRS axis was between -30 degrees and -90 degrees.

For diagnosing old MI in the presence of LBBB, the so-called "Cabrera's sign" [8], a notching in the first 0.04 seconds in duration in the ascending limb of the S wave in lead V3 or V4, and "Chapman's sign" [9] a notching ≥ 0,05 sec in the ascending limb of the R wave in D1, aVL or V6.

In this study, the notching ≥ 0,05 sec in the ascending or descending limb of the S or R wave in every lead was evaluated and compared between groups. The amplitudes of the first (R) and second (R') positive deflection in LBBB morphology were also compared with each other in V5,V6, D1 and aVL leads.

The statistical package SPSS for Windows (Release 11.5, SPSS Inc) was used for statistical analysis. The categorical variables were expressed as a percentage and analyzed by chi-square statistics. The continuous variables were expressed as mean and analyzed by one-way ANOVA, and post hoc Tukey tests were also used to further investigate these differences, as appropriate. Associations were assessed by Pearson correlation coefficient. Receiver operating characteristic (ROC) analysis on different ECG variables was performed to assess the sensitivity and specificity of different threshold values to distinguish between NILVD and ILVD. A 2-tailed P value of 0.05 or less was considered significant.

## Results

At the files of all (2567) patients in the hospital's computer archives, 85 patients with LBBB had LVEF > 40% on echocardiographic data, and was excluded the study. The study population included 98 patients; 49 patients had NILVD (22 female; age: 59 ± 12 years), 49 patients had ILVD (20 female; age: 65 ± 11 years).

In comparison with the voltage indices, the voltage of R wave in V6 was statistically higher in NILVD group compared to ILVD group (p: 0.03). The other voltage indices had no statistically differences between the groups (Table 2).

The presence of the depression of the ST-J point by more than 0.2 mV in V6 was more frequent in NILVD group compared to ILVD group (p= 0.001) (Table 3). No significant differences were found the other ECG criteria reported in Table 3. The presence of an abnormal Q waves in DI, aVL and V6 could not be compared statistically due to low sample volume with Q wave in groups.

In comparison to the height of the first (R) and second (R') positive deflections in V5-V6-D1-aVL, no statistically difference was found between the groups (Table 4).

While the notching in the ascending or descending limb of the S wave in V1-4 leads was more frequent in ILVD group (p: 0.03); the other notching localizations in LBBB morphology were not shown statistically difference (Table 5). ROC analysis was assayed for voltage of R wave in V6 and showed no substantial discriminative power.

## Discussion

It is well known that the detection of ECG-based diagnoses such as acute myocardial infarction or left ventricular hypertrophy in the presence of LBBB is problematic due to alterations in the timing of ventricular depolarization [10-13]. In addition, the differentiating role of ECG features in the presence of LBBB is debatable for differentiating ILVD from NILVD. However, various ECG criteria of LBBB have been the object of many studies [14-16]. Momiyama et al. in their study evaluating the ECG characteristics of NILVD  has been reported that the patients with NILVD commonly showed the ECG signs of left ventricular hypertrophy evaluated by Sokolow's criterion [15]. In their another study [16], whereas the voltage of R wave in lead V6 was the highest in NILVD group as similar to our study results, the voltage of R wave waves in leads DI, DII, and DIII were lowest in NICMP group. RV6 voltage of 15 mm or more was present in 78% of NILVD patients compared with 11% of CAD patients (P < 0.001). The R waves in leads I, II, and III (RI, RII, RIII) were also low in NILVD. Therefore, all voltage ratios of RV6/RI, RII, RIII were the highest in NILVD. In particular, the ratio of RV6 over the maximum R wave in leads I, II, and III (RV6/Rmax) was significantly higher in the NILVD  group compared with the CAD and control groups, and correlated with the degree of left ventricular dilatation and inversely with ejection fraction. This ratio of 3 or more was present in 61% of the NILVD patients but in none of the CAD patients or normal subjects. They also reported that abnormal Q waves were seen in 69% of CAD patients and in 26% of NILVD  patients. Abnormal Q waves in leads DII, DIII, aVF, or V2-V4 were present in 61% of CAD patients compared with 4% of NILVD patients. In our study, abnormal Q wave could not be evaluated due to low sample in groups.

Bayes-Genis et al [17]. have been reported that the voltages of precordial leads V2, V3  and sum of (V1 + V2 + V3) voltages were significantly more prominent in patients with NILVD. Although they found that the sensitivity and specificity of V3 voltage >2100 microV on surface ECG in the presence of LBBB to identify a left ventricular dysfunction of nonischemic origin were 85 and 73%, respectively. In present study, the voltage indices were not showed any differences between the groups.

The present study has several limitations. Firstly, the number of patients reported in this study was a bit larger than a number of other studies performed in the modern era, however, these findings should be validated prospectively in a larger cohort, and the effect of the criteria on patient care should also be examined. Secondly, it is well known that differentiation between ILVD and NILVD could be difficult in some patients. There are many patients who have CAD, or have percutaneous coronary interventions performed and an LVEF of < 40% due to nonischemic reasons (e.g., due to hypertension). Similar to this, some patients with CAD may have severely depressed ventricular function beyond that expected for the amount of CAD, such as a patient with single (non-LAD) disease and a markedly reduced ejection fraction. Although the definite distinction could be complicated in such as circumferences, even single vessel disease was included as ILVD in current study .The another on is that, in this study, the ECG taken at the day of the coronary angiography was used. Amplitudes could be notoriously variable in the precordial leads of serial ECGs of the same patient, but, the present study was not compared the serial ECGs changes of the same patient.

In conclusion, the present study showed that, although some ECG findings were found to be useful, ECG criteria had poor value in differentiating ILVD from NILVD  in the presence of complete LBBB.

## Figures and Tables

**Table 1:**
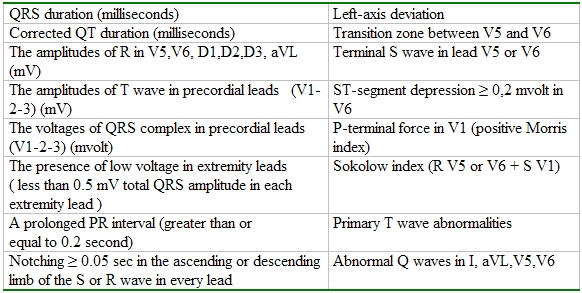
Electrocardiographic criteria analyzed in LBBB

**Table 2:**
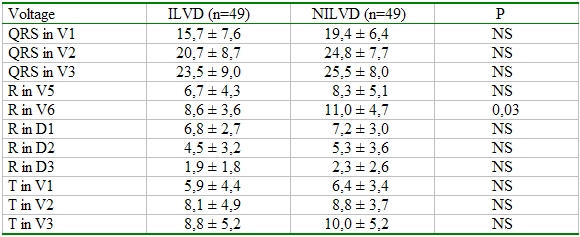
Comparison of voltages indices between groups

Vol: voltage. Voltage values are expressed as  0,1 millivolt (mvolt). NS: nonsignificant

**Table 3:**
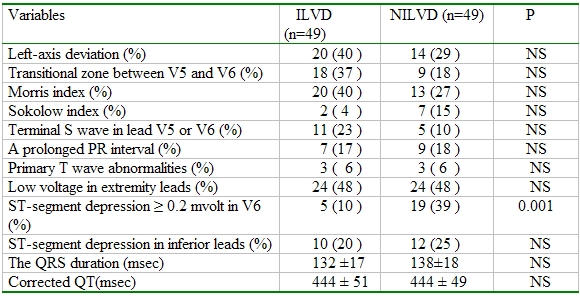
The comparison of the presence of some ECG criteria between groups

NS: nonsignificant

**Table 4:**
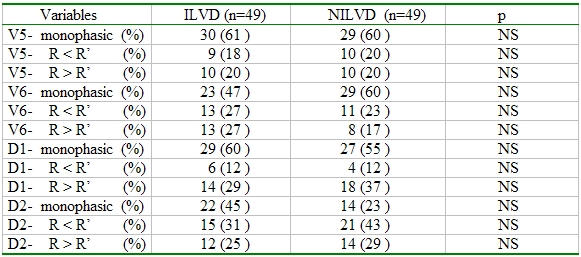
Comparision of first (R) and second (R') positive deflection of LBBB morphology in V5-V6-D1-aVL

NS: nonsignificant

**Table 5:**
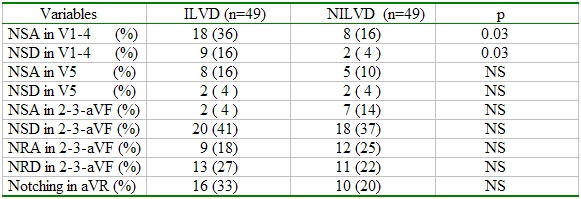
Comparision of the notching localizations in LBBB morphology

NSA: notching in the ascending limb of the S wave NSD: notching in the descending limb of the S wave NRA: notching in the ascending limb of the R wave NRD: notching in the descending limb of the R wave NS: nonsignificant
